# Feline cognitive dysfunction as a model for Alzheimer’s disease in the research of CBD as a potential treatment—a narrative review

**DOI:** 10.1186/s42238-020-00054-w

**Published:** 2020-12-17

**Authors:** Lilach Zadik-Weiss, Sivan Ritter, Vered Hermush, Nethanel Asher, Avi Avital, Reuven Or

**Affiliations:** 1 Amsterdam, The Netherlands; 2 Tel Aviv, Israel; 3 Department of Geriatrics and Skilled Nursing, Laniado Medical Center, Netanya, Israel; 4 Rappaport Faculty of Medicine, Technion—Israel Institute of Technology, Haifa, Israel; 5 The Ella Lemelbaum Institute for Immuno-Oncology, Sheba Medical Center, Ramat Gan, Israel; 6 Behavioral Neuroscience Lab, Department of Neuroscience, The Rappaport Faculty of Medicine, Technion—Israel Institute of Technology, Haifa, Israel; 7 Bone Marrow Transplantation, Cancer Immunotherapy & Immunobiology Research Center, Hadassah-Hebrew University Medical Center, Jerusalem, Israel

**Keywords:** Feline cognitive dysfunction, Alzheimer’s disease, Cannabidiol, Reverse translational medicine, One Health

## Abstract

With the improvement in modern medicine, the world’s human and feline (*Felis catus*, the domestic cat) population is aging. As the population grows older, there is an increase of age-related diseases, such as Alzheimer’s disease in humans and feline cognitive dysfunction in felines, which shares many similarities with Alzheimer’s disease. They both result in cognitive decline and lack effective treatments. In light of their pathological similarities, both occur at old age, and as domestic cats share the human environment and risk factors (cats are considered an indicator to the effect of environmental contaminants on humans as they share exposures and diseases), cats have the potential to be a spontaneous model for Alzheimer’s disease. Classic animal models in many cases fail to predict the results in humans, and a natural model can lead to better prediction of results, thus being both time and cost-effective. The feline disease can be researched in trials that could be simultaneously clinical trials for cats and preclinical trials for humans, also referred to as *reverse translational medicine*. As both maladies lack effective medical intervention, new potential treatments are merited. Cannabidiol (CBD) is a promising agent that may improve the life of these patients, as it was shown to potentially treat several of the pathologies found in both conditions. yet there is a need for further research in order to establish the benefits and safety of CBD to both human and feline patients.

## Background

According to global estimates in 2019, there were over 50 million people suffering from dementia, and by 2050 that number is expected to increase to 152 million (Alzheimer’s Disease International 2020). Alzheimer’s disease (AD) is the most common cause of dementia in humans. The disease causes a gradual cognitive decline appearing at early stages as mild short-term memory loss and is easily compensated. However, the more advanced stages affect everyday life and require support in basic everyday functions (Alzheimer’s Association 2019). The owned cat population is also aging thanks to improved nutrition and medical veterinary care. The aging cat population displays behavioral changes which can be attributed to cognitive dysfunction (Karagiannis and Mills 2014). Feline cognitive dysfunction (FCD) is a disorder in cats which causes a decline in the aging cat’s cognitive abilities, without any known underlying medical reason (Karagiannis and Mills 2014; Gunn-Moore et al. 2015). FCD causes behavioral changes, of which the most common are disorientation, change in social interactions with humans or other pets, changes in sleep wake patterns, house soiling, and excessive vocalization, especially at night (Landsberg et al. 2012; Gunn-Moore et al. 2007). Many cats over the age of 11 present some signs of this disorder, while most are symptomatic from 16 and over. Since these behavioral disorders are difficult for owners to live with and compromise the cat’s quality of life, they are often a cause of euthanasia (Karagiannis and Mills 2014). The diagnosis of this syndrome, in living patients, is based solely on exclusion of other medical or behavioral issues, for example, hyperthyroidism, systemic hypertension, diabetes mellitus (DM), deafness, osteoarthritis (or other causes of chronic pain), and brain tumors (Landsberg et al. 2012), and is complicated as these symptoms are frequently attributed to normal aging of the cat (Bellows et al. 2016). Similar changes can be seen in aging humans and dogs (Landsberg et al. 2012).

The available treatments for humans can only treat the disease’s symptoms as there are currently no targeted drugs that stop or reverse disease progression (Salomone et al. 2012). In cats, there are no registered medications to treat FCD and varying success has been observed from off-label options. Additionally, older cats and especially cats suffering from cognitive dysfunction do not tolerate either handling, or medication or hospitalization well (due to the stress involved). Exploring treatments that can be administered in their food would be beneficial.

Several animal models have shown cannabidiol (CBD) has potential to treat and reverse the changes seen in the brain in AD, although further research to support this is necessary (Watt and Karl 2017).

Alzheimer’s disease and feline cognitive dysfunction severely impair the patient’s health and quality of life and impose a significant burden on their caretakers. As the affected population is expected to grow, there is an urgent need to find treatments that can stop and reverse the deterioration of both human patients and the animal population suffering from similar conditions.

## Pathological changes in Alzheimer’s disease and in feline cognitive dysfunction

The accumulation of extracellular plaques is a pathognomonic pathological finding in AD. These are misfolded β-amyloid (Aβ) proteins and tau tangles which are the result of hyperphosphorylated tau proteins. Additional findings present in the AD brain are oxidative stress, microglial activation, neuronal loss, and chronic inflammation in the brain which is linked to the neurodegeneration present in AD (Morales et al. 2014; Sarlus and Heneka 2017). Treating the inflammation had beneficial effects in animal models (Lim et al. 2000). In cats, common pathologies found in the brains of patients diagnosed with cognitive dysfunction include decreased cerebral blood flow, free radical damage, and neuronal loss. Additionally, aging domestic cats may spontaneously develop both β-amyloid and tau pathologies similar but not identical to those seen in human AD (Fiock et al. 2020; Chambers et al. 2015). Several studies found β-amyloid aggregates in the aging feline brain (Gunn-Moore et al. 2007; Gołaszewska et al. 2019; Gunn-Moore et al. 2006), although the aggregates present in human and feline patients are different (Gunn-Moore 2011). Other studies found that cats display tau aggregates in their brain with a similar spread pattern as found in human AD patients (Fiock et al. 2020; Gunn-Moore 2011; Poncelet et al. 2019; Youssef et al. 2016) and with shared characteristics (Head et al. 2005). The chronic brain damage leads to a disease similar to the human Alzheimer’s disease (Gunn-Moore et al. 2015; Gunn-Moore 2011). While cats frequently develop spontaneously occurring Tau pathologies, many other animal species, including dogs, rarely express these pathologies (Chambers et al. 2015). Additionally, cats that show signs of behavioral dysfunction tend to also have Aβ plaques (Cummings et al. 1996).

## Animal models for Alzheimer’s disease: laboratory animals versus pets with naturally occurring disease

A cross species approach to disease research can advance the understanding of AD and FCD and benefit patients of all species (Devinsky et al. 2018). There is an abundance of published research concerning AD based on laboratory animals, such as transgenic mice (Rosenmann et al. 2008). Although, when the potential treatments are tested in the clinical trial phases, as required by good clinical practice (GCP) guidelines in humans, the results are not as promising and are sometimes hazardous (Götz and Ittner 2008). Laboratory animal models, such as transgenic mice, frequently fail to predict the outcome of the tested potential treatments in humans (Polson and Fuji 2012). Many drugs that seemed promising in animal models did not succeed in clinical trials (Watt and Karl 2017). In fact, it has been concluded that the available animal models do not reflect the human AD fully or accurately (Birch et al. 2014). Examples in AD research include anti-inflammatory drugs that have beneficial effects in animal models and not in human clinical trials (Grammas 2011). Additionally, anti-Aβ vaccination that was found to prevent the accumulation of amyloid deposits in transgenic mice caused severe neurological complications in some of the human patients who received it (Foster et al. 2009).

There are many possible reasons for the failure to translate results from animal models to humans. The animal experiments were carried out with laboratory animals, mostly transgenic that may manifest other deficits. Relating to limited construct, content, ecological, and face validity, animal models are held in a closed regulated laboratory environment and are not exposed to similar environmental contaminants as human patients, which is of high significance in a disease that is also influenced by environmental factors (Cannon and Greenamyre 2011). Therefore, laboratory animal models cannot accurately model natural occurring diseases in a natural home environment. None of the available animal models can represent AD completely since none of them is able to replicate all the features of the human disease; moreover, very few models have both amyloid plaques and tau tangles (Drummond and Wisniewski 2017). Transgenic animal models do not express all the factors present in the human disease, including pathological changes and disease progression; they also do not reflect the etiology of the human disease which is multifactorial and includes genetic and environmental risk factors (Franco and Cedazo-Minguez 2014). Transgenic animal models usually model only a single pathological feature and lack factors that mimic the environmental factors together with the cognitive deficits and with the pathological features. The ideal model should reflect the entire etiology as well as the disease progression (Franco and Cedazo-Minguez 2014).

Moreover, transgenic animal models usually include genetically modified young animals, thereby ignoring an imperative factor in AD which is the aging factor. Domestic cats with naturally occurring disease are also aging as the symptoms are present in cats older than 11 years old and more commonly over 16. Additionally, the transgenic models for AD are based on the familial forms of AD which account only for a small percentage of the AD patients (Epis et al. 2010).

A better model for human disease can be found in owned pets with naturally occurring disease which share the human environment (Ritter et al. 2020). Pets are considered sentinels and bioindicators for environmental contaminants effects on humans due to their shared habitat, simultaneous exposure, and similar disease spectrum (Pastorinho 2020; Beck et al. in press). According to the One Health Initiative, there is a strong link between the health of humans, domestic animals, and the environment. Humans and domestic animals share the same environment (including environmental contaminant), common stressors, and many genetic traits (Christopher 2015). One Health promotes human and animal health through integrative study across all animal species (Gibbs 2014). Since cats suffering from FCD are exposed to the same environment as their owners, the disease mechanism is naturally occurring and at an old age, they make a good model. These studies can prove beneficial to human and veterinary patients (Schneider et al. 2018). The brain lesions have common pathological characteristics in humans and cats, and it has been suggested that cats are a natural model of AD; they demonstrate lesions with morphological and biochemical spontaneous changes that are comparable to the human AD lesions and they do so in a shorter lifespan (Head et al. 2005).

It is worth mentioning that dogs are also potentially natural models for AD in humans as many old dogs also suffer from similar cognitive dysfunction (Prpar Mihevc and Majdič 2019).

Clinical feline trials can be preclinical trials for humans, and medication can be simultaneously developed in a time and cost-effective process. These are known today as *reverse translational medicine* (Schneider et al. 2018). Additionally, they have the potential to promote animal welfare by avoiding unnecessary use of laboratory animals. The feline disease shares many similarities with AD and although the diseases are not identical we suggest that the similarities AD and FCD share warrant exploring the feline disease as a model to the human disease.

Cats and dogs share pathological changes with the human AD and share the human environment which makes them both good candidate models for naturally occurring AD (Takeuchi et al. 2008). While dogs share many similarities with the human AD, cats are one of the only species which displays naturally occurring tau pathologies (Chambers et al. 2015). It was shown that aging cats share three important similarities with human AD; they have spontaneous development of Aβ dispositions, and they have taupathies and neuronal loss which shares distribution and characteristics with AD (Klug et al. 2020). Moreover, it has been shown that there is a correlation between the neuronal loss and cognitive dysfunction in aging cats (Takeuchi et al. 2008). These facts make cats, at the very least a complementary model to dogs in researching naturally occurring AD.

## CBD as potential treatment for Alzheimer’s disease

Among the various functions of cannabidiol are anti-inflammatory properties, demonstrated in vitro and in vivo (in rats) (Cassano et al. 2020; Esposito et al. 2011) and antioxidant properties (Esposito et al. 2011; Pellati et al. 2018) *in vitro* (Hartsel et al. 2019; Kim et al. 2019). Additionally, CBD was found to inhibit the hyperphosphorylation of tau proteins in vitro (Esposito et al. 2006), which, as mentioned, is present in AD human patients and in some FCD patients. CBD has also been shown to increase the cerebral blood flow *in vivo* (in mouse models after stroke) (Sultan et al. 2017), attenuate the neurotoxicity of the β-amyloid protein accumulation in vitro and in vivo (in mice), modulate microglial cell function (Martín-Moreno et al. 2011), and reverse cognitive deficits in transgenic animal models (in mice) (Cheng et al. 2014). Thus, CBD has the potential to counter many of the pathological processes in AD (Vallée et al. 2017). There is a need for more research regarding the benefits, risks, and doses for CBD treatment in human patients and especially in elderly human patients (Beedham et al. 2020). The safety of the treatment needs to be evaluated for chronic use (Iffland and Grotenhermen 2017), and potential drug interactions should be evaluated as many elderly patients need to be treated for concurrent conditions (Alsherbiny and Li 2018). Additionally, there is a need to research the usage of CBD to treat FCD patients. Combining the research could benefit feline and human patients and save resources such as time and funds. Clinical evidence in treating dementia or AD with CBD is scarce, we conducted an electronic search in “PubMed” and “Google Scholar” for published articles, in English, published in the years 2005 to September 2020, using the key words “Dementia + Cannabidiol + clinical trials”, “Alzheimer’s + Cannabidiol + clinical trials”, “Dementia + Cannabidiol”, and “Alzheimer’s + Cannabidiol”, and their references and citations. We did not find articles regarding clinical trials in treatment of AD or dementia with CBD. Additionally, a few recent comprehensive reviews that addressed the treatment of AD and dementia with cannabinoids did not review clinical trials with treatment of dementia or AD with CBD (Beedham et al. 2020; Inglet et al. 2020; Cooray et al. 2020). A published case communication demonstrated treatment of an 81-year-old man that suffered from dementia after several cardiovascular events and presented with drowsiness, difficulty keeping his eyes open, inability to maintain eye contact for more than a few seconds, inability to speak, difficulties to communicate, and severe spasticity. He was treated with CBD, starting with 3 daily drops of CBD oil and after approximately 7 days the dose was increased to 4 daily drops of CBD oil. A few days after commencing treatment, his alertness and responsiveness improved and he was able to say a few words. A month after beginning the treatment, he remained more alert and responsive, continued to say a few words, and regained his ability to make eye contact for more than a few seconds, and his spasticity decreased. No side effects were reported (Hermush and Ore 2019).

## CBD and feline patients

There is a shortage of literature regarding the usage of CBD in domestic cats and the pharmacokinetics and safety must be established. A comprehensive review of the existing literature on veterinary cannabinoid medicine does not mention feline research in the field (Hartsel et al. 2019). One research tested the pharmacokinetics and safety of a single daily dose of CBD in a small group of cats for 84 days and found that it was absorbed after oral administration and that the safety was satisfactory, although monitoring of liver enzymes is necessary (Deabold et al. 2019). Research with larger numbers of cats is necessary to establish the safety and effectiveness of the treatment. Additionally, a double blind study treating domestic feline cognitive dysfunction patients in significant numbers is necessary to determine the usefulness of this treatment for feline patients and its potential use for human patients.

## Conclusions

Classic animal models fail to predict the outcome of new treatments more than they succeed. This causes inefficient use of resources and compromises animal welfare without promoting human or animal health. We suggest, in accord with the One Health Initiative, that a better model for human disease can be found in domestic pets, with naturally occurring diseases who share the human environment so the disease mechanisms and features have a higher chance of simulating the human equivalent. Moreover, we suggest that these trials should be conducted as clinical veterinary trials to develop treatment for the parallel conditions in domestic pets, thus enhancing animal welfare, promoting better veterinary care, and saving resources by simultaneously developing treatments for humans and pets. We find feline cognitive dysfunction a promising model for human Alzheimer’s disease. Therefore, we suggest that conducting a clinical trial in aging domestic cats, for researching the benefits of CBD for both conditions, can promote the treatment of these two difficult conditions in humans and pets.

## Data Availability

Not applicable.

## Abbreviations

AD: Alzheimer’s disease
FCD: Feline cognitive dysfunction
CBD: Cannabidiol
